# Work productivity in the office and at home during the COVID‐19 pandemic: A cross‐sectional analysis of office workers in Japan

**DOI:** 10.1111/ina.12913

**Published:** 2021-07-23

**Authors:** Wataru Umishio, Naoki Kagi, Ryo Asaoka, Motoya Hayashi, Takao Sawachi, Takahiro Ueno

**Affiliations:** ^1^ Department of Architecture and Building Engineering School of Environment and Society Tokyo Institute of Technology Meguro‐ku Tokyo Japan; ^2^ Department of System Design Engineering Faculty of Science and Technology Keio University Yokohama Kanagawa Japan; ^3^ Faculty of Engineering Division of Architecture Hokkaido University Sapporo Hokkaido Japan; ^4^ The Building Center of Japan Chiyoda‐ku Tokyo Japan; ^5^ Building Research Institute Tsukuba Ibaraki Japan

**Keywords:** COVID‐19, PM_2.5_, productivity, work environment, work from home, work in the office

## Abstract

The coronavirus disease 2019 (COVID‐19) pandemic has drastically changed work styles and environments. Given the coexistence of work in the office and work from home (WFH) in the future, studies are needed to identify ways to increase productivity when working in both places. We conducted a questionnaire survey and environment measurements of 916 workers in 22 offices across 2 weeks in November–December 2020 in Japan. While average workdays at the offices decreased from 4.9 to 3.9 days/week, those at homes increased from 0.1 to 1.1 days/week due to COVID‐19, indicating an increase in the relative importance of WFH. Compared to the office, the satisfaction rate was lower for lighting, spatial, and information technology (IT) environments, but higher for thermal, air, and sound environments at home. Although it was easier to concentrate on work and to refresh at home, workers experienced challenges associated with business communication from home. Meanwhile, in the office, satisfaction with COVID‐19 countermeasures was significantly associated with work productivity. Furthermore, lower PM_2.5_ concentration was associated with greater satisfaction with COVID‐19 countermeasures, indicating that reducing PM_2.5_ may increase satisfaction with COVID‐19 countermeasures and work productivity. We expect these findings will help improve work productivity in the New Normal era.

**Practical implications**

- At home, the spatial, sound, and information technology (IT) environment were important for work productivity.
- In the office, in addition to the spatial, sound, and IT environment, satisfaction with COVID‐19 countermeasures was important for work productivity.
- Lower PM_2.5_ concentration was associated with greater satisfaction with COVID‐19 countermeasures.
- At present, CO_2_ concentration is considered as an important index of poorly ventilated closed spaces which is one of the risk factors for COVID‐19.
- PM_2.5_ may be an important index of workers’ satisfaction with COVID‐19 countermeasures and productivity.

## 1 INTRODUCTION

Since 2020, the coronavirus disease 2019 (COVID‐19) pandemic has threatened public health worldwide, and caused drastic changes to work styles and work environments. With regard to work style, COVID‐19 has accelerated the recent trend to work from home (WFH). According to the International Labour Organization, only 7.9% of the world's workforce worked from home on a permanent basis prior to the COVID‐19 pandemic.^1^ As a result of government‐imposed lockdowns and declarations of a state of emergency in 2020, however, WFH became a more common practice for workers worldwide. Currently, although workers are gradually moving back into office buildings, large numbers continue to WFH.

COVID‐19 has also changed the work environment. Many workplaces have introduced countermeasures based on the modes of transmission of severe acute respiratory syndrome coronavirus 2 (SARS‐CoV‐2): physical distancing as a countermeasure for droplet transmission, and cleaning and disinfecting surfaces as a countermeasure for contact transmission. In addition, evidence on airborne transmission is increasing,^2^, ^3^, ^4^, ^5^, ^6^, ^7^ with the World Health Organization (WHO)^8^ and the Centers for Disease Control and Prevention (CDC)^9^ recognizing airborne transmission as a mode of transmission for SARS‐CoV‐2. This has led to the issuance^10^, ^11^, ^12^ and review^13^ of guidelines for heating, ventilation, and air‐conditioning (HVAC) systems during the COVID‐19 pandemic in various countries as countermeasures for airborne transmission. Such changes in the operation of HVAC systems may lead to changes in the work environment.

Furthermore, Japan enforced the “Work Style Reform Law” in April 2019, indicating the urgent need to enhance productivity. Given the drastic changes to work styles and work environments during compared to before the COVID‐19 pandemic, it would be interesting to examine productivity in current work styles and work environments. While many studies have examined the relationship between the work environment in the office and productivity before the COVID‐19 pandemic,^14^, ^15^, ^16^, ^17^, ^18^, ^19^ few studies have been conducted since the pandemic began. Several papers have recently focused on the effects of WFH.^20^, ^21^ However, given the likely coexistence of work in the office and WFH in the New Normal era, it is necessary to examine the relationship between work environment and productivity both in the office and at home, in particular, which work environments (eg, lighting, thermal, air, sound, spatial, and information technology (IT) environment) are strongly associated with productivity.

We conducted a survey on work style, work environment and productivity in the offices and at home during the COVID‐19 pandemic. The purpose of this study was to investigate the link between different work styles and work environments and productivity during the COVID‐19 pandemic, and to explore ways to improve productivity in the New Normal era.

## 2 METHODS

### 2.1 Study design

**FIGURE 1:**
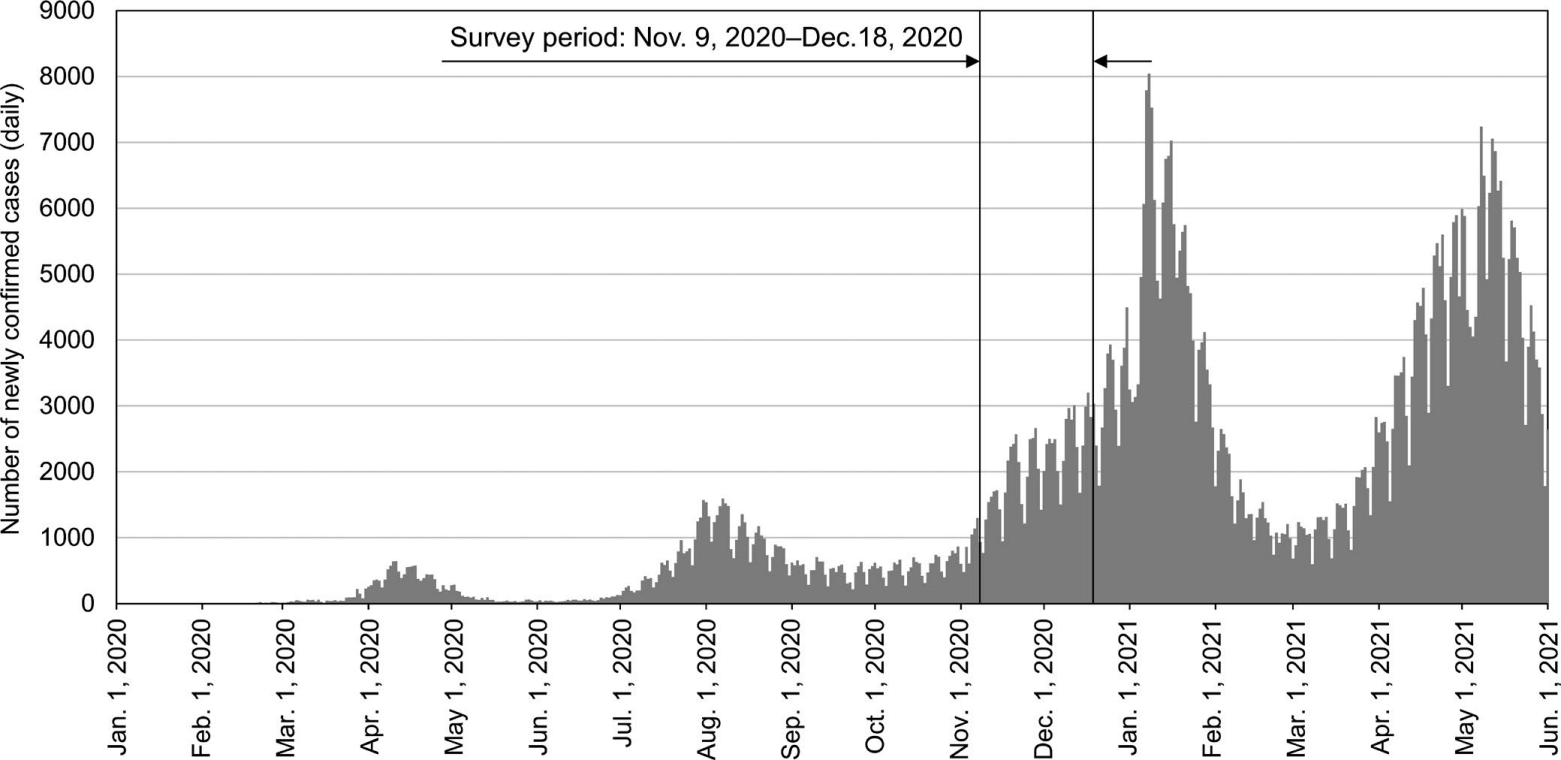
Trend in the number of newly confirmed cases of COVID‐19

This survey was conducted across 2 weeks in November and December 2020 (during the COVID‐19 pandemic). Figure 1 shows the trend in the number of newly confirmed cases of COVID‐19.^22^ The survey was carried out in the beginning of a third wave (average 2120 (range: 770–3206) cases/day). We recruited building operators and office workers from 22 buildings of 18 companies in Japan. The detailed sample size in each building is shown in Table S1. The study protocol and informed consent procedure were approved by the Tokyo Institute of Technology's Human Subjects Research Ethics Review Committee (approval No. 2020063). All participants indicated an intention to participate in the survey beforehand.

### 2.2 Questionnaire survey

Two types of questionnaires were administered, one to building operators and the other to office workers. The questionnaire items are shown in Table S2. Briefly, the questionnaire for building operators covered building information, HVAC systems, and their maintenance. Questionnaire items about system maintenance were taken from several guidelines on COVID‐19 issued by American,^10^, ^23^, ^24^ European,^11^ and Japanese organizations.^12^ The questionnaire for workers covered individual attributes, work style before/during COVID‐19, office/home work environment, productivity (concentration on work and creative tasks, ability to relax and refresh with ease, and ease of communication), lifestyle and mental health. We used the same questions and answers to the work style before and during COVID‐19 to compare 2 periods equally. Similarly, we did to the office and home work environment. Participants responded to items about the office/home work environment and productivity on a 7‐point scale and 5‐point scale, respectively. Lifestyle was investigated using the International Physical Activity Questionnaire (IPAQ) short form^25^, ^26^ and the Athens Insomnia Scale (AIS).^27^ Mental health was examined using the K6^28^ and Work Functioning Impairment Scale (WFun).^29^

### 2.3 Office environment measurements

Indoor temperature and humidity, CO_2_ concentration, and PM_2.5_ mass concentration were used as office environment factors. Indoor temperature, relative humidity, and CO_2_ concentration were measured at 5‐min intervals for 2 weeks using a logger (TR‐76Ui; T&D Corp.) placed on the desk of a representative worker. At the same time, PM_2.5_ mass concentration was measured at 1‐min intervals using a logger placed next to TR‐76Ui (PMT‐2500; Komyo Rikagaku Kogyo K.K.). These loggers were kept away from heat‐generating or aerosol‐generating devices such as printers because printers are a source of PM_2.5_ in the office. They were also placed away from direct sunlight because it may affect measurements of temperature and data measured using laser light scattering methods. Office environment factors from 9 a.m. to 5 p.m. on weekdays were averaged and used in the analyses to take the regular work hours into consideration. Outdoor temperature and humidity values were obtained from the closest local meteorological observatory, and outdoor PM_2.5_ from the closest local Atmospheric Environmental Regional Observation System (AEROS) to each building.

### 2.4 Statistical analysis

Proportions of related samples were compared using the marginal homogeneity test, an extension of McNemar's test. Multiple linear regression analysis was used to examine the association between satisfaction with the office/home environment and productivity. Model A to E included productivity as a dependent variable and satisfaction with the environment as an independent variable. Models were adjusted for age; gender; work type (engineer or not); sleep condition (AIS score); and physical activity (IPAQ Short).

The association between office environment factors and satisfaction with COVID‐19 countermeasures was analyzed using a multilevel linear regression model. The dependent variable was satisfaction (on a 7‐point scale) with COVID‐19 countermeasures. A two‐level random intercept model was used in which office worker‐level variables (age, gender, work type (engineer or not), work style (work days at the office) and lifestyle (AIS score)) were nested within office‐level variables (temperature, relative humidity, CO_2_ concentration and PM_2.5_ mass concentration). Office worker‐level variables were centered around the mean for each office, while office‐level variables were centered around the overall mean. Regression coefficients were estimated using the maximum likelihood method. All *p* values were two‐sided, and a two‐sided *p* value less than 0.05 was considered statistically significant. All analyses were performed using SPSS Ver. 26 (SPSS Inc.,).

## 3 RESULTS

### 3.1 Baseline characteristics of workers, work styles and work environment

Table 1 shows the characteristics of 916 workers. About 80% of workers answered the questionnaire in the office. Age of workers ranged from <30 years to ≥60 years, and three‐quarters of the workers were men. Two‐thirds of the workers were technical staff in research and development or design and engineering. Regarding health literacy, 95.1% of workers wore masks during work, 77.0% of workers always washed their hands after arriving at the office, and about 47.5% always measured their body temperature before leaving for the office.

**TABLE 1:** Characteristics of office workers

Variable	Number	(%)
Place where workers answered the questionnaire		
Office	732	(79.9)
Home	165	(18.0)
Others	19	(2.1)
Age		
<30 years	172	(18.7)
30s	237	(25.9)
40s	204	(22.3)
50s	250	(27.3)
≥60 years	53	(5.8)
Gender		
Male	707	(77.2)
Female	209	(22.8)
Work type		
Office clerk	60	(6.6)
Administration, accounting, human resources	35	(3.8)
Material sourcing and procurement	5	(0.5)
Management, planning	76	(8.3)
Research and development	513	(56.0)
Design, engineering	107	(11.7)
Sales	72	(7.9)
Production/manufacturing management	17	(1.9)
Others	30	(3.3)
Wearing mask		
Both working and commuting	681	(74.5)
Only working	188	(20.6)
Only commuting	39	(4.3)
None	6	(0.7)
Washing hands after arriving at the office		
Always (without fail)	703	(77.0)
Sometimes	178	(19.5)
Never	32	(3.5)
Checking body temperature before leaving for the office		
Always (without fail)	433	(47.5)
Sometimes	239	(26.2)
Never	240	(26.3)

**FIGURE 2:**
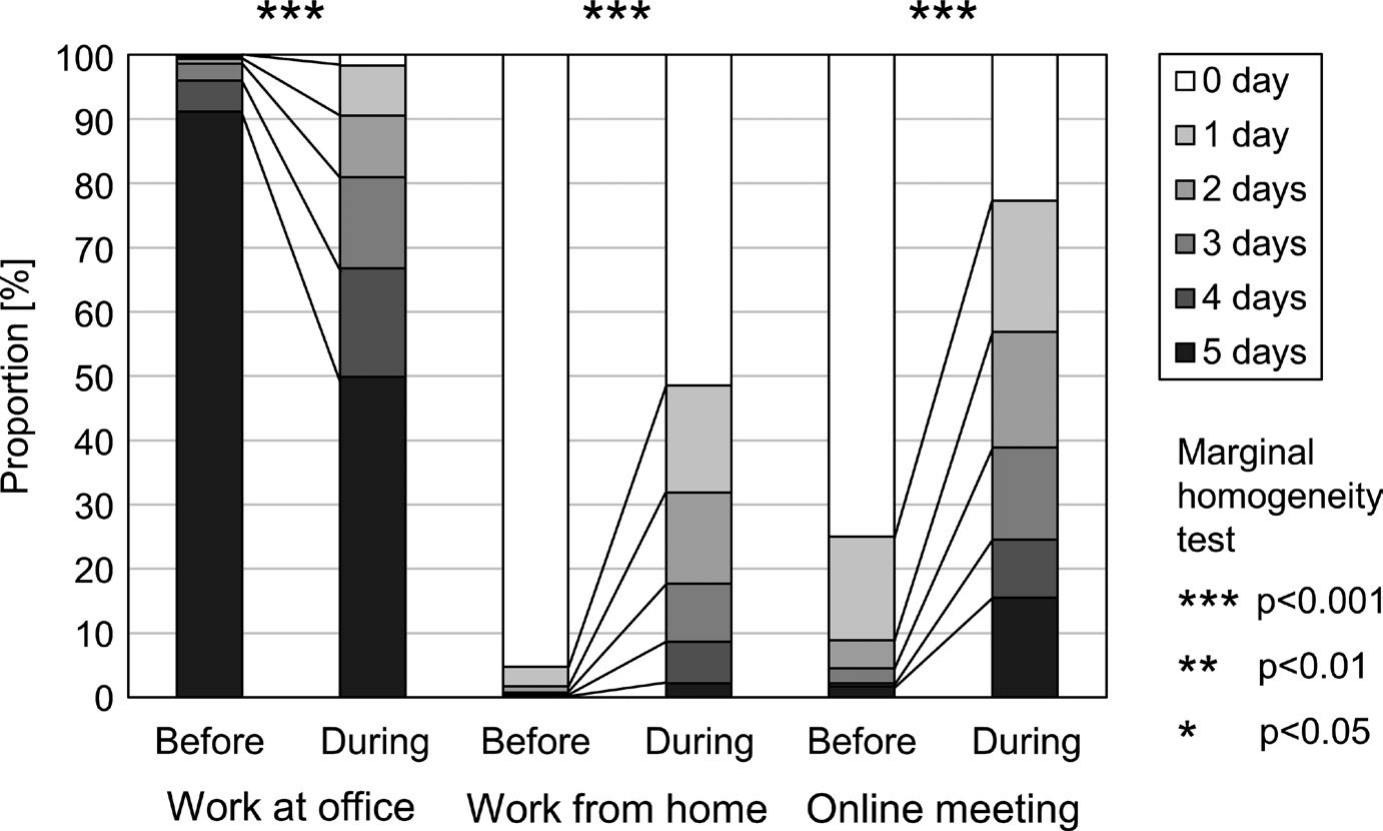
Work style before and during COVID‐19

Data from 777 workers who had worked in their current workplace for ≥1 year were used to compare work styles before and during COVID‐19. Average workdays at the office decreased from 4.9 to 3.9 days/week, while those at home increased from 0.1 to 1.1 days/week. Average number of days engaging in online meetings increased from 0.4 to 2.1 days/week (Figure 2).

**FIGURE 3:**
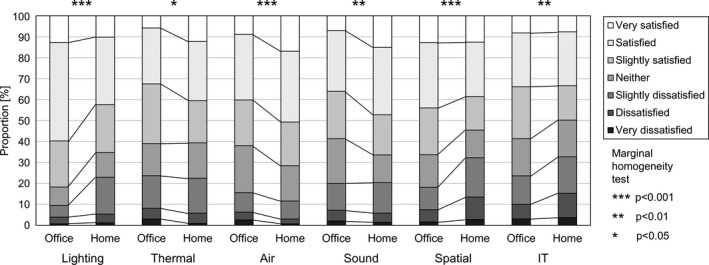
Satisfaction with the work environment in the office and at home

**FIGURE 4:**
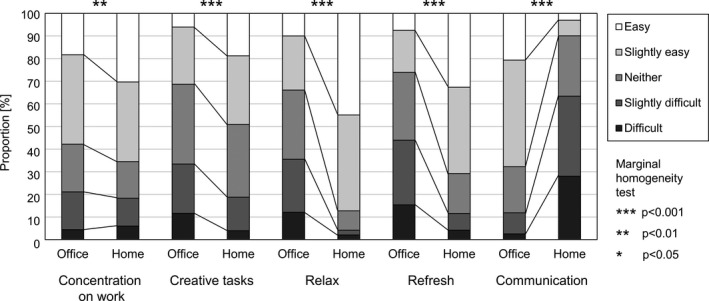
Productivity in the office and at home

Data from 432 workers who worked ≥1 day/week both in the office and at home were used to compare environmental satisfaction and productivity. Compared to the office, significantly fewer workers were satisfied with the lighting (illumination of the desk), spatial (room size), and IT environment (Internet connection speed and stability) at home (Figure 3). In contrast, more workers were satisfied with the thermal (temperature, humidity, etc.), air (stagnation), and sound environment (noise) at home. In terms of productivity, although it was easier to concentrate on work and creative tasks, and to relax and refresh at home than in the office, workers at home experienced challenges associated with business communication (Figure 4).

### 3.2 Office environment measurements

**FIGURE 5:**
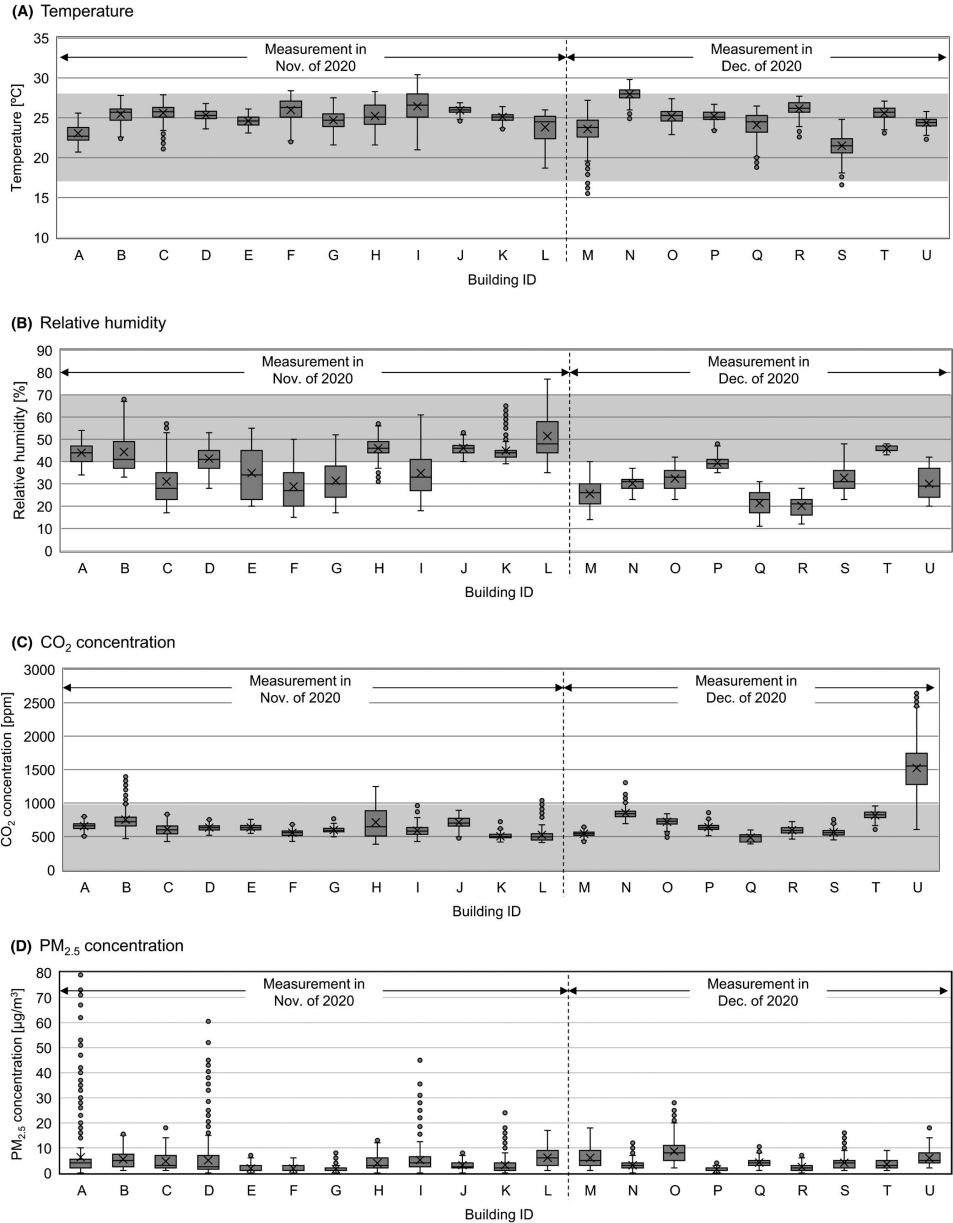
Indoor environmental factors in office buildings during the COVID‐19 pandemic Measurements were conducted in 21 buildings except for the Building ID: V. Gray areas indicate the air environment requirements of the Act on Maintenance of Sanitation in Buildings. Data obtained from 9 a.m. to 5 p.m. on weekdays were analyzed. Circles indicate outliers. When the length of the whisker was 1.5 times longer than the inter‐quartile range, the value was defined as an outlier

Figure 5 shows the indoor temperature, relative humidity, CO_2_ concentration, and PM_2.5_ mass concentration in 21 buildings; measurements were not conducted in 1 building. Average indoor temperature was 24.9℃, and all buildings met the air environment requirement (17–28℃) of the Act on Maintenance of Sanitation in Buildings. Average relative humidity was 36.1%, and 13 out of 21 buildings had levels below 40% (air environment requirement is 40–70%). Average CO_2_ concentration was 666 ppm, and only 1 building had an average reading above 1000 ppm (air environment requirement is ≤1000 ppm). Average PM_2.5_ mass concentration was 4.3 µg/m^3^.

### 3.3 Association between satisfaction with office/home environment and productivity

The results of multiple linear regression analyses are shown in Table 2A–E. The most important environmental factors for productivity were different between the office and home. In terms of concentration on work, the sound environment was the most important in the office, while spatial environment was the most important at home. For creative tasks, spatial environment was the most important both in the office and at home. In terms of the ability to relax and refresh with ease, spatial environment was the most important in the office, while the air environment was the most important at home. Spatial and lighting environments in the office, and spatial and IT environments at home were closely correlated with ease of communication. Furthermore, satisfaction with COVID‐19 countermeasures in the office was significantly associated with concentration on work, creative tasks, and the ability to relax and refresh with ease.

**TABLE 2:** Multiple linear regression analysis of satisfaction with the office/home environment and productivity

(A) Objective variable: Concentration on work						
Independent variable	Office			Home		
	*β*	Standardized *β*	*p* value	*β*	Standardized *β*	*p* value
Lighting environment	0.06	0.06	0.056	0.07	0.09	0.075
Thermal environment	0.02	0.03	0.439	−0.04	−0.05	0.325
Air environment	0.02	0.02	0.545	0.04	0.04	0.457
Sound environment	0.25	0.33	<0.001	0.10	0.13	0.012
Spatial environment	0.07	0.10	0.009	0.20	0.28	<0.001
IT environment	0.06	0.09	0.007	0.08	0.10	0.017
COVID−19 countermeasures	0.10	0.12	<0.001	−	−	−

(B) Objective variable: Creative tasks						
Independent variable	Office			Home		
	*β*	Standardized *β*	*p* value	*β*	Standardized *β*	*p* value
Lighting environment	0.02	0.02	0.524	0.05	0.07	0.186
Thermal environment	0.03	0.03	0.378	0.04	0.06	0.312
Air environment	0.04	0.05	0.200	0.04	0.04	0.445
Sound environment	0.10	0.14	<0.001	0.03	0.03	0.510
Spatial environment	0.16	0.22	<0.001	0.18	0.28	<0.001
IT environment	0.06	0.08	0.015	0.05	0.07	0.099
COVID−19 countermeasures	0.11	0.13	<0.001	−	−	−

(C) Objective variable: ability to relax with ease						
Independent variable	Office			Home		
	*β*	Standardized *β*	*p* value	*β*	Standardized *β*	*p* value
Lighting environment	0.09	0.09	0.006	−0.02	−0.04	0.472
Thermal environment	0.01	0.02	0.658	0.04	0.06	0.278
Air environment	0.03	0.03	0.438	0.12	0.18	0.001
Sound environment	0.11	0.14	<0.001	0.06	0.10	0.056
Spatial environment	0.13	0.16	<0.001	0.09	0.16	0.002
IT environment	0.08	0.10	0.002	0.03	0.06	0.153
COVID−19 countermeasures	0.13	0.14	<0.001	−	−	−

(D) Objective variable: Ability to refresh with ease						
Independent variable	Office			Home		
	*β*	Standardized *β*	*p* value	*β*	Standardized *β*	*p* value
Lighting environment	0.05	0.05	0.141	−0.02	−0.03	0.566
Thermal environment	0.04	0.05	0.177	0.06	0.08	0.123
Air environment	0.08	0.09	0.031	0.19	0.23	<0.001
Sound environment	0.08	0.10	0.005	0.05	0.06	0.210
Spatial environment	0.13	0.16	<0.001	0.10	0.16	0.002
IT environment	0.04	0.05	0.143	0.05	0.07	0.096
COVID−19 countermeasures	0.08	0.09	0.010	−	−	−

(E) Objective variable: Ease of communication						
Independent variable	Office			Home		
	*β*	Standardized *β*	*p* value	*β*	Standardized *β*	*p* value
Lighting environment	0.11	0.13	0.001	0.05	0.06	0.250
Thermal environment	0.02	0.03	0.549	0.08	0.10	0.068
Air environment	0.04	0.06	0.204	−0.06	−0.08	0.181
Sound environment	0.03	0.04	0.262	−0.00	−0.01	0.916
Spatial environment	0.11	0.15	<0.001	0.13	0.19	<0.001
IT environment	0.03	0.04	0.267	0.08	0.13	0.005
COVID−19 countermeasures	0.05	0.06	0.098	−	−	−

Note

Adjusted for age, gender, work type (engineer or not), sleep condition (AIS score) and physical activity (IPAQ Short).

### 3.4 Satisfaction with COVID‐19 countermeasures and quality of the indoor environment

**FIGURE 6:**
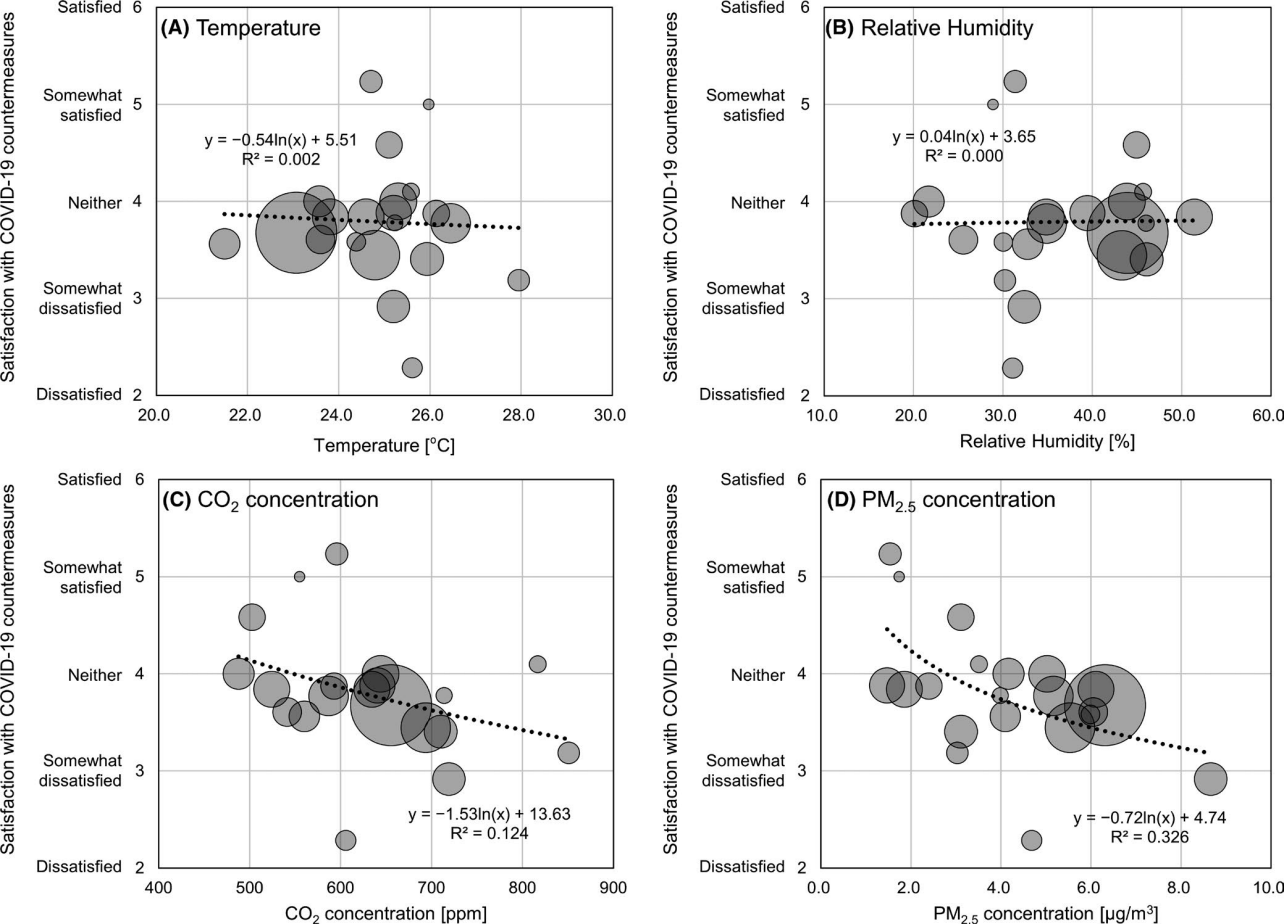
Indoor environmental factors and satisfaction with COVID‐19 countermeasures each circle shows data from each building. The size of each circle indicates the number of respondents in each building. The log function has been selected among the linear, square, power, and exponential function based on the R^2^ and *p* value of curve fitting analyses by SPSS (Table S3)

Our findings suggested that COVID‐19 countermeasures may affect productivity in the office. We therefore analyzed the association between office environment measurements and satisfaction with COVID‐19 countermeasures, excluding data from the building with abnormally high CO_2_ concentrations (Building U in Figure 5). Temperature and relative humidity were not associated with satisfaction with COVID‐19 countermeasures. In contrast, lower CO_2_ and PM_2.5_ levels were correlated with higher satisfaction with COVID‐19 countermeasures (Figure 6). A similar trend was observed in the multilevel linear regression model after adjusting for confounders (Table 3). Although CO_2_ concentration was not significantly correlated (*p *= 0.061), PM_2.5_ mass concentration was significantly correlated with satisfaction with COVID‐19 countermeasures (*p *= 0.009). For reference, we show the concrete COVID‐19 countermeasures conducted in 20 buildings except for 2 buildings whose information could not be collected or disclosed (Table S4).

**TABLE 3:** Multilevel linear regression analysis of satisfaction with COVID‐19 countermeasures

Independent variable		Univariate model			Multivariate model^a^		
		*β*	(95%CI)	*p* Value	*β*	(95%CI)	*p* value
Office‐level variable							
Temperature	[°C]	−0.035	(−0.231, 0.161)	0.709	−0.068	(−0.186, 0.173)	0.935
Relative humidity	[%RH]	0.003	(−0.028, 0.033)	0.851	0.014	(−0.009, 0.038)	0.213
CO_2_ concentration	[ppm]	−0.002	(−0.005, 0.000)	0.085	−0.003	(−0.005, 0.000)	0.061
PM_2.5_ concentration	[µg/m^3^]	−0.156	(−0.278, −0.034)	0.016	−0.160	(−0.271, −0.049)	0.009

^a^ Adjusted for office worker‐level variables such as age, gender, work type (engineer or not), work style (work days in the office) and sleep condition (AIS score).

## 4 DISCUSSION

### 4.1 Summary of findings

This study analyzed the association between productivity and work environment in the office and at home during the COVID‐19 pandemic (November–December 2020). Cross‐sectional analyses of 916 workers in 22 buildings showed that (a) compared to their work style before COVID‐19, the average number of workdays in the office decreased from 4.9 to 3.9 days/week, while those at home increased from 0.1 to 1.1 days/week; (b) compared to the office, the satisfaction rate was lower for lighting, spatial, and IT environments, but higher for thermal, air, and sound environments at home; (c) all buildings met the air environment requirements for temperature (17–28℃) and all except one met the air environment requirements for CO_2_ concentration (≤1000 ppm); (d) satisfaction with COVID‐19 countermeasures in the office was significantly associated with productivity; and (e) lower PM_2.5_ mass concentration was significantly correlated with higher satisfaction with COVID‐19 countermeasures (*p *= 0.009).

### 4.2 Work style and work environment during the COVID‐19 pandemic

In 2019, only 4.8% of workers had engaged in WFH practices, compared to 49.2% as of November/December 2020. Similarly, 25.1% of workers engaged in online meetings in 2019, compared to 77.3% in November/December 2020. Work styles have drastically changed and the relative importance of WFH has increased; both offices and homes are now essential workplaces.

Workers showed lower satisfaction with lighting, spatial, and IT environments at home than in the office. Given that the home is classically a place for rest, standard light fixtures and Internet infrastructure may be unsuitable for work. Furthermore, according to an international comparison, the total floor area of houses in Japan is small,^30^ thus making it difficult to establish a dedicated space for work at home. Conversely, workers showed greater satisfaction with the thermal environment at home because they were in direct control of the temperature. Satisfaction with the air and sound environments was also higher at home. One possible reason for this is that indoor air quality problems (eg, droplet nuclei and human bioeffluents) and noise (eg, small talk) from other workers may not bother workers because only their close family members are at home.

Regarding office work environments, almost all buildings examined in the present study met the requirements for CO_2_ concentration. In contrast, about 30% of buildings exceeded the recommended CO_2_ concentration of 1000 ppm before COVID‐19, according to a report on air environment in 2017.^31^ This difference may be due to the purposive increase in the ventilation air volume to reduce CO_2_ concentrations as a COVID‐19 countermeasure for airborne transmission. Concurrently, all buildings in the present study met the requirement for indoor temperature, while about 30% of buildings were outside the required range of 17–28℃ in 2017.^31^ The combination of the two factors, an increase in the amount of outdoor air and maintaining the indoor temperature within the appropriate range, indicates that inefficient energy use occurred as a countermeasure for airborne transmission of SARS‐CoV‐2.

### 4.3 How to improve satisfaction with COVID‐19 countermeasures

The present results showed that satisfaction with COVID‐19 countermeasures was significantly associated with productivity in the office. This suggests that increasing satisfaction with COVID‐19 countermeasures may increase productivity in the office. Furthermore, the present study also indicated that a lower PM_2.5_ mass concentration was strongly correlated with higher satisfaction with COVID‐19 countermeasures. Although PM_2.5_ is invisible to the human eye, countermeasures for reducing PM_2.5_ such as installing medium efficiency air filters and air purifiers may increase satisfaction with COVID‐19 countermeasures and work productivity. One of the possible reasons is that the Society of Heating, Air‐Conditioning and Sanitary Engineers of Japan has conducted awareness‐raising activities on the air filter and the air purifier since the beginning of the pandemic,^12^ resulting in that workers in Japan were concerned about the indoor air quality. At present, CO_2_ concentration is considered as an important index of poorly ventilated closed spaces which is one of the risk factors for COVID‐19. Similarly, PM_2.5_ may be an important index in terms of satisfaction with COVID‐19 countermeasures and productivity during the pandemic. Focusing only on the COVID‐19, we guess that the association between PM_2.5_ and satisfaction with COVID‐19 countermeasures/productivity might weaken when the world turns to be normal. However, given that the pandemic occurred again and again in history, we believe our findings is useful when it comes to the future pandemic.

### 4.4 How to increase work productivity in the new normal era

The present study shows that both the type of work and work environment must be considered to ensure sufficient productivity in a workplace. As shown in Figure 4, it was easier to concentrate on work at home than in the office. However, as shown in Table 2, the quality of spatial, sound, and IT environments such as adequate room size, quietness, and Internet speed were necessary for concentration. While it was easier to communicate with others in the office than at home, smoother communication required higher quality spatial and lighting environments. Therefore, both the type of work and the quality of the work environment need to be considered when selecting an appropriate workplace to facilitate productive work. Interestingly, a third type of work environment outside the home and office, such as a satellite office and co‐working space, has attracted more attention recently,^32^ and may be another solution for those whose homes and offices do not reach a certain level as work environments.

### 4.5 Study limitations

This study has several limitations. First, because the majority of workers in this study were engineers, the present results may have some bias. For example, the type of work and work style of engineers differ from those of non‐engineers; thus, the present results may not be applicable to non‐engineers. We also compared the distributions of age and gender in the present survey with those in the Labor Force Survey in Japan.^33^ The proportion of workers ≥60 years was 5.8 vs 21.3%, the proportion of male workers was 77.2 vs 55.7% (the present vs the national survey). The workers in the present survey were biased toward younger age group and male. Therefore, the applicability of the findings should be considered cautiously. Second, the work productivity was measured based on the questionnaire, the results might be biased by personal preferences for working in the office or at home. A future study using objective indices such as the number of typing mistakes or sleepiness measured by eye‐tracking devices is necessary to eliminate the bias. Third, we only surveyed workers for a short period (November–December 2020) of the COVID‐19 pandemic. Given that the COVID‐19 pandemic is continually evolving, the present results should be interpreted in the context of the study period. In particular, work styles may change again once the majority of the population has been vaccinated. We suggest that additional research is needed after the completion of mass vaccination among the general public.

## 5 CONCLUSIONS

This survey of 916 workers in 22 buildings in Japan showed that the COVID‐19 pandemic has caused substantial changes to work styles (eg, adoption of WFH practices), leading to the coexistence of work in the office and WFH (3.9 days/week vs 1.1 days/week). Both at home and in the office, spatial, sound, and IT environments were important for work productivity. However, spatial and IT environments at home and sound environment in the office had room for improvement compared with one another. Furthermore, in the office, satisfaction with COVID‐19 countermeasures, in addition to spatial, sound, and IT environments, was important for work productivity. Besides, lower PM_2.5_ concentration was associated with greater satisfaction with COVID‐19 countermeasures, indicating that appropriate management of PM_2.5_ may increase work productivity as a result of improving satisfaction with COVID‐19 countermeasures. We expect these findings will help improve work productivity in the New Normal era.

## CONFLICT OF INTEREST

No conflict of interest has been declared by the authors.

## AUTHOR CONTRIBUTIONS

**Wataru Umishio:** Conceptualization‐Equal, Data curation‐Lead, Formal analysis‐Lead, Investigation‐Lead, Methodology‐Lead, Validation‐Lead, Visualization‐Lead, Writing‐original draft‐Lead. **Naoki Kagi:** Conceptualization‐Equal, Investigation‐Supporting, Methodology‐Supporting, Resources‐Lead, Validation‐Supporting, Writing‐review & editing‐Lead. **Ryo Asaoka:** Data curation‐Supporting, Formal analysis‐Supporting, Investigation‐Supporting, Validation‐Supporting, Visualization‐Supporting, Writing‐review & editing‐Supporting. **Motoya Hayashi:** Conceptualization‐Equal, Project administration‐Lead, Supervision‐Lead, Writing‐review & editing‐Supporting. **Takao Sawachi:** Funding acquisition‐Lead, Project administration‐Supporting, Supervision‐Supporting, Writing‐review & editing‐Supporting. **Takahiro Ueno:** Funding acquisition‐Supporting, Project administration‐Supporting, Supervision‐Supporting, Validation‐Supporting, Writing‐review & editing‐Supporting.

### PEER REVIEW

The peer review history for this article is available at https://publons.com/publon/10.1111/ina.12913.

## Supporting information

Table S1‐S4
