# Supplementary material for: Work productivity in the office and at home during the COVID‐19 pandemic: A cross‐sectional analysis of office workers in Japan
Source: Indoor Air. 2021 Jul 23;32(1):e12913. doi: 10.1111/ina.12913 (PMC8446952; doi:10.1111/ina.12913)
Supplement: Supplementary file 1 — Table S1‐S4 [file INA-32-0-s001.docx]

**Supplementary Material**

**Supplement to:**

**Work productivity in the office and at home during the COVID-19 pandemic: a cross-sectional analysis of office workers in Japan**

Wataru Umishio, Naoki Kagi, Ryo Asaoka, Motoya Hayashi, Takao Sawachi, Takahiro Ueno

**Appendix S1: Supplement tables and figures**

Table S1. Detailed number of respondents in 22 buildings and 18 companies

Table S2. Outline of questionnaire survey for building operators and office workers

Table S3. Curve fitting analyses to the relationship between PM_2.5_ and satisfaction with COVID-19 countermeasures

Table S4. COVID-19 countermeasures in office buildings

Table S1. Detailed number of respondents in 22 buildings and 18 companies

| Company ID | Building ID | Number of respondents |
| --- | --- | --- |
| 1 | A | 222 |
| 2 | B | 84 |
| 3 | C | 14 |
| 4 | D | 46 |
| 5 | E | 44 |
| 6 | F | 4 |
| 7 | G | 17 |
| 8 | H | 11 |
| 9 | I | 53 |
| 10 | J | 37 |
|  | K | 24 |
| 11 | L | 48 |
| 12 | M | 28 |
|  | N | 16 |
|  | O | 36 |
| 13 | P | 42 |
|  | Q | 34 |
| 14 | R | 24 |
| 15 | S | 32 |
| 16 | T | 10 |
| 17 | U | 12 |
| 18 | V | 78 |

Table S2. Outline of questionnaire survey for building operators and office workers

| Target | Classification | Item |
| --- | --- | --- |
| Operator | Building information | Location, year of completion of construction, building structure, total floor area |
|  | Ventilation and AC systems | Heat source, HVAC system, ventilation volume, automatic control of ventilation volume, humidification, return and outdoor air filter |
|  | Maintenance | Air filter, monitoring of environmental parameters, cleaning, exchanging air before/after using rooms |
| Worker | Individual attribute | Age, gender, work type, satisfaction with work, commuting, health literacy (wearing mask, washing hands, checking body temperature) |
|  | Workstyle before/with COVID-19 period | Work in office, online meeting, work from home, productivity, overtime work hours |
|  | Office and stay-home work environment | Satisfaction with lighting, thermal, air, sound, spatial, and IT environment,  Satisfaction with COVID-19 countermeasures,  Productivity (concentration on work and creative tasks, ability to relax and refresh with ease, and ease of communication),  anxiety of infection to COVID-19,  COVID-19 countermeasures† |
|  | Lifestyle and health condition | Sleep (AIS),  Physical activity (IPAQ Short),  Mental health (K6),  Work functioning impairment scale (WFun) |

AC, air conditioning; HVAC, heating, ventilation, and air conditioning; IT, information technology; IPAQ, International Physical Activity Questionnaire; AIS, Athens Insomnia Scale; K6, Kessler Psychological Distress Scale (6-item version); WFun, Work Functioning Impairment Scale

† answered by a representative worker of each building

Table S3. Curve fitting analyses to the relationship between PM_2.5_ and satisfaction with COVID-19 countermeasures

| Function | R^2^ value | P value |
| --- | --- | --- |
| Linear | 0.290 | 0.012 |
| Square | 0.307 | 0.037 |
| Log | 0.326 | 0.007 |
| Power | 0.281 | 0.013 |
| Exponential | 0.256 | 0.019 |

Table S4. COVID-19 countermeasures in office buildings

| Variable | Number | (%) |
| --- | --- | --- |
| Countermeasures conducted by building operators |  |  |
| Increasing the amount of outdoor air | 10 | (50.0) |
| Maintaining negative pressure in the toilet | 6 | (30.0) |
| Checking the AC filter condition | 10 | (50.0) |
| Replacing the AC filter | 8 | (40.0) |
| Installing UV lamp to the AC system | 0 | (0.0) |
| Disinfection of high-touch surfaces | 12 | (60.0) |
| Checking the ventilation system works properly | 14 | (70.0) |
| Monitoring temperature, humidity, and CO_2_ regularly | 15 | (75.0) |
| Cleaning the AC system (duct, grille, etc.) | 10 | (50.0) |
| Changing the air before and after using a room | 11 | (55.0) |
| Keeping relative humidity within 40-70% | 11 | (55.0) |
| Keeping CO_2_ concentration under 1000ppm | 10 | (50.0) |
| Countermeasures conducted in workplaces |  |  |
| Building plan and furniture |  |  |
| Using outdoor area for working or eating | 2 | (10.0) |
| Physical distancing by partitions or furniture | 15 | (75.0) |
| Layout (making empty desks or staggering desks) | 12 | (60.0) |
| One-way traffic flow or separating entrances | 1 | (5.0) |
| Setting up personal workstations | 4 | (20.0) |
| Installing active furniture and furnishing | 1 | (5.0) |
| Scheme of sign |  |  |
| Signage of post hygiene, cleaning, and sanitizing | 1 | (5.0) |
| Signage of recommended spacing among workers | 7 | (35.0) |
| Signage of the required occupant limits | 5 | (25.0) |
| Signage of recommendation of wearing a mask | 10 | (50.0) |
| Signage of recommendation of hand washing | 10 | (50.0) |
| Signage of recommendation of natural ventilation | 6 | (30.0) |
| Countermeasures for the contact transmission |  |  |
| Contactless body temperature measurement | 8 | (40.0) |
| Contactless doors and equipment | 4 | (20.0) |
| Installing alcohol disinfectant | 20 | (100.0) |
| Installing disposal paper towel | 6 | (30.0) |
| Countermeasures for the airborne transmission |  |  |
| Natural ventilation by opening windows | 10 | (50.0) |
| Visualizing temperature, humidity, and CO_2_ | 4 | (20.0) |
| Using air purifiers | 5 | (25.0) |
| Upgrading IT environment |  |  |
| Maintaining Internet infrastructure | 15 | (75.0) |
| Installing online meeting software (Zoom, Teams, etc.) | 19 | (95.0) |
| Installing communication software (Slack, etc.) | 7 | (35.0) |
| Providing monitors, earphones, and microphones | 10 | (50.0) |
| Providing wearable devices | 2 | (10.0) |

AC, Air conditioning; UV, ultraviolet; IT, information technology
